# Dose-dependent regulation of horizontal cell fate by Onecut family of transcription factors

**DOI:** 10.1371/journal.pone.0237403

**Published:** 2020-08-13

**Authors:** Michaela Kreplova, Andrea Kuzelova, Barbora Antosova, Lucie Zilova, Herbert Jägle, Zbynek Kozmik

**Affiliations:** 1 Laboratory of Transcriptional Regulation, Institute of Molecular Genetics of the Czech Academy of Sciences, Prague, Czech Republic; 2 Laboratory of Eye Biology, Division BIOCEV, Institute of Molecular Genetics of the Czech Academy of Sciences, Vestec, Czech Republic; 3 Department for Ophthalmology, University Hospital Regensburg, Regensburg, Germany; National Eye Centre, UNITED STATES

## Abstract

Genome duplication leads to an emergence of gene paralogs that are essentially free to undergo the process of neofunctionalization, subfunctionalization or degeneration (gene loss). Onecut1 (Oc1) and Onecut2 (Oc2) transcription factors, encoded by paralogous genes in mammals, are expressed in precursors of horizontal cells (HCs), retinal ganglion cells and cone photoreceptors. Previous studies have shown that ablation of either *Oc1* or *Oc2* gene in the mouse retina results in a decreased number of HCs, while simultaneous deletion of *Oc1* and *Oc2* leads to a complete loss of HCs. Here we study the genetic redundancy between *Oc1* and *Oc2* paralogs and focus on how the dose of Onecut transcription factors influences abundance of individual retinal cell types and overall retina physiology. Our data show that reducing the number of functional Oc alleles in the developing retina leads to a gradual decrease in the number of HCs, progressive thinning of the outer plexiform layer and diminished electrophysiology responses. Taken together, these observations indicate that in the context of HC population, the alleles of Oc1/Oc2 paralogous genes are mutually interchangeable, function additively to support proper retinal function and their molecular evolution does not follow one of the typical routes after gene duplication.

## Introduction

The retina represents a neural tissue in the eye and transmits information about the light stimulus to target locations in the brain [1]. Neuroretina is composed of six types of neurons: rod and cone photoreceptors, horizontal cells (HCs), bipolar cells (BCs), amacrine cells (ACs), retinal ganglion cells (RGCs); and of one glial cell type: Müller cells (MGCs) [2–4]. Müller glia cells maintain the retinal homeostasis and define the retinal boundaries and polarity. Therefore, loss of MGCs would cause defects in retinal lamination and even result in retinal degeneration [5]. In the mature retina, retinal cell are arranged in three layers: outer nuclear layer (ONL) containing rod and cone photoreceptors, inner nuclear layer (INL) composed of HCs, BCs, MGCs, and ACs and ganglion cell layer (GCL) consisting of RGCs and displaced ACs [2–4]. Synaptic connections are organized into two separated layers: thin outer plexiform layer (OPL) and inner plexiform layer (IPL). OPL is composed of HC and BC projections and photoreceptor terminals [6, 7], whereas IPL is formed by a branching pattern of AC processes that synapse with RGCs and BCs [8]. During retinogenesis, particular retinal cell types originate from a common pool of multipotent retinal progenitor cells (RPCs). The choice of individual retinal cell fates is determined by cooperation of extrinsic cues of the changing environment and by intrinsic factors, mainly basic helix-loop-helix (bHLH) and homeodomain class of transcription factors [9–11]. Order in which particular retinal cell types are generated is conserved across species, RGCs are generated first, while rods, BCs and Müller glia are generated last [12]. Generation of particular cell types proceeds in two overlapping waves [12]. Early born cell types are generated mostly during embryonal stage, whereas late born cell types are generated mostly postnatally. Early born cell types are RGCs, HCs, ACs, cone photoreceptors and late born cell types are BCs, MGCs and rod photoreceptors [13].

Onecut (Oc) family transcription factors were identified as Pax6 downstream-acting factors [11]. Pax6 plays a crucial role in multipotency of retinal progenitor cells and is required for initiation of the retinal differentiation program [14]. Inactivation of Pax6 results in failure of specific retinal cell fate determination [2, 14, 15]. Oc transcription factors contain a homeodomain and a cut domain, both important for DNA binding [16]. Originally, cut homeodomain proteins were characterized as products of *Drosophila cut* genes [17]. In mammals, Oc transcription factors are represented by three members: Onecut 1 (Oc1), Onecut2 (Oc2) and Onecut3 (Oc3) [18]. Oc transcription factors were characterized as transcription factors controlling cell differentiation in the liver, pancreas, immune system [16, 19–23], and CNS [19, 24, 25]. Oc1 was originally called hepatocyte nuclear factor 6 (HNF-6) [19, 26, 27], described as a transcriptional activator of liver promoter of the 6-phosphofructo-2-kinase (*pkf-2*) gene [17]. Constitutitive inactivation of Oc1 caused high mortality, 75% of mice died before weaning between postnatal days 1–10 (P1-P10) as a result of liver defects [28]. Oc1 plays important role in endocrine development. Development of pancreas was disrupted in Oc1^-/-^ and mice were diabetics [28]. Oc transcription factors are expressed in the developing mouse retina and have overlapping expression patterns, resulting in their functional redundancy. Oc3 is expressed together with Oc1 and Oc2, but at a relatively low level [7, 29]. It was suggested that transcription factors Oc1 and Oc2 take part in the birth, differentiation and subtype generation (with the exception of rods having only one subtype) of each retinal cell type [30]. The expression of Oc1 and Oc2 starts at the early stage of retinal development in precursors of RGCs, HCs and cone photoreceptors [7, 11, 29, 31]. In the mature retina, Oc1 and Oc2 transcription factors are expressed only in HCs [11]. Previous studies have found that Oc1 and Oc2 are crucial for HC development and maintenance [7, 11, 30–32]. Simultaneous action of Oc1 and Otx2 promotes the fates of cone photoreceptors and horizontal cells and represses rod photoreceptors [33]. Horizontal cell fate starts from RPCs, which co-express Pax6 and Foxn4. Foxn4 controls AC and HC genesis. The AC genesis is independent of Pax6 [34], in contrast to HC genesis, which is governed by cooperation of Pax6 and Foxn4. Pax6 with Foxn4 stimulate expression of Oc1 and Oc2 [11] and Foxn4 activates expression of Ptf1a [35]. Cells expressing only Ptf1a adopt the AC fate, while cells expressing Ptf1a together with Oc1 and Oc2 assume the HC fate [30]. Ptf1a and Oc1 with Oc2 are required for the regulation of Lim1 and Prox1 in HCs, Ptf1a in early and Oc1 with Oc2 in late developmental stages [11]. Deletion of either the *Oc1* or *Oc2* gene results in a decreased number of HCs and reduced OPL in mouse retinas [7, 31, 32]. Deletion of both Oc1 and Oc2 leads to a complete loss of HCs and absence of OPL [11, 31]. The absence of OPL causes ONL and INL to fuse and form a single layer. In addition, Oc1/Oc2 double mutants appeared to have diminished numbers of RGCs, BCs, MGCs, starburst ACs and cones [11, 31].

Current studies did not investigate double heterozygosity involving *Oc1* and *Oc2* loci in mice, or mice with a single remaining functional allele of the *Oc1* or *Oc2* gene. Our data analysis of all possible *Oc1*/*Oc2* genotypes indicate that the abundance of HCs is highly dependent on the dose of Oc proteins. Other retinal cell types were significantly affected only in mice with simultaneous deletion of both Oc transcription factors. The only exception were the cones, which were also reduced in *Oc1*^*-/-*^ and *Oc1*^*-/-*^*; Oc2*^*+/-*^ genotypes. The retinal intensity-response functions measured by flash electroretinogram recording were also increasingly impaired in mice with the reduced *Oc* gene dose. Taken together, this study suggests that HCs are exquisitely sensitive to the level of Onecut transcription factors that are required for proper retinal function.

## Materials and methods

### Experimental animals

Previously described mouse strains: BAC transgenic *mRx-Cre* [36], *Oc1*^fl/fl^ [37] and *Oc2*^*+/-*^ [23] were used in this study. *Oc1* was specifically deleted in the retina by *mRx-Cre*, *Oc2* was deleted constitutively. Housing of mice and in vivo experiments were performed in compliance with the European Communities Council Directive of 24 November 1986 (86/609/EEC) and national and institutional guidelines. Animal care and experimental procedures were approved by the Animal Care Committee of the Institute of Molecular Genetics (no. 71/2014). Mice were euthanized by cervical dislocation. This work did not include human subjects.

### Hematoxylin-Eosin (H&E) staining

Whole eyes from mice at stage P18 were removed, fixed in 4% paraformaldehyde (w/v) at 4°C overnight, dehydrated and embedded in paraffin. Eyes were sectioned at 5 μm by fully motorized rotary Leica RM2255 microtome. Sections were dewaxed, stained with H&E and mounted in 80% glycerol. Images were obtained by light microscopy imaging using a Nikon Diaphot 300 inverted microscope and processed by Fiji-ImageJ and FastStone Image Viewer.

### Immunohistochemistry

Deparaffinized sections of P18 mice were incubated for 20 minutes in citrate buffer (10mM, pH 6.0) at 98°C in a steam bath for antigen retrieval, washed with PBS and permeabilized by PBS with 0.1% Tween 20, blocked with 10% BSA and incubated with primary antibody at 4°C overnight. The following primary antibodies were used: mouse anti-Ap2α (Santa-Cruz, 12726X, 1:3000), mouse anti-Calbindin-D-28K (Sigma-Aldrich, clone CB-955, C9848, 1:2500), mouse anti-Lim1/2 (DSHB, clone 4F2-c, 1:300), rabbit cone arrestin (Millipore, AB15282, 1:1000), rabbit anti-Pax6 AF1 (1:3000), rabbit Sox9 (Millipore, 1:500), rabbit anti-Tbr2 (Abcam, ab23345, 1:500), sheep anti-Chx10 (ThermoFisher Scientific, PA1-12565, 1:500). Next day, sections were washed with PBS, incubated with fluorescent secondary antibody Alexa Fluor 488 or 594 (Life Technologies, 1:500) for 1 hour at room temperature, washed with PBS, counterstained with DAPI (1 μg/ml) and mounted in mowiol (4–88; Sigma).

### Cell counting

Fluorescence images were acquired with a Leica SP5 confocal microscope using 40x objective, zoom factor 2 for calbindin, Lim1/2, Ap2α, Sox9, Chx10, Pax6 and Tbr2 and with 63x objective for cone arrestin. Images were then processed by Fiji-ImageJ and Adobe Photoshop. DAPI and marker-positive retinal cells were counted in whole acquired single eye fields of the central retinal sections, obj. 40x 193,75 x 193,75 μm, obj. 63x 246,03 x 246,03 μm. Only horizontal cells were counted in whole retinal sections. For each mouse minimum of 4 sections were counted. Number of P18 mice that were analyzed is specified in graphs separately for each staining. Statistical significance was determined by Welch´s ANOVA test in GraphPad Prism. If differences between the means were statistically significant (p<0.05), Dunnett´s T3 multiple comparison test was performed. In cases where less than 3 mice were counted, statistical significance was not performed for these samples.

### Electroretinogram recording

ERGs were recorded according to a procedure described previously [38, 39]. Additionally, responses to flickering stimuli (intensity 0.5 log cds/m2) with frequencies ranging from 4 to 25 Hz were recorded. Flicker response waveforms were analyzed by means of a fast Fourier transform to calculate response magnitude and phase and to estimate signal significance. All analyses and plotting were carried out with R 3.6.0 and ggplot 3.2.0. Mice used for analysis were 15–17 weeks old, in total: 6 wt mice, 6 Oc1^+/-^; Oc2^+/-^ mice, 6 Oc1^-/-^ mice, 3 Oc2^-/-^ mice, 3 Oc1^-/-^; Oc2^-/-^ mice, 6 Oc1^-/-^; Oc2^+/-^ mice and 4 Oc1^+/-^; Oc2^-/-^ mice.

## Results

### Failure of OPL development in Oc-deficient retinas

**Fig 1:**
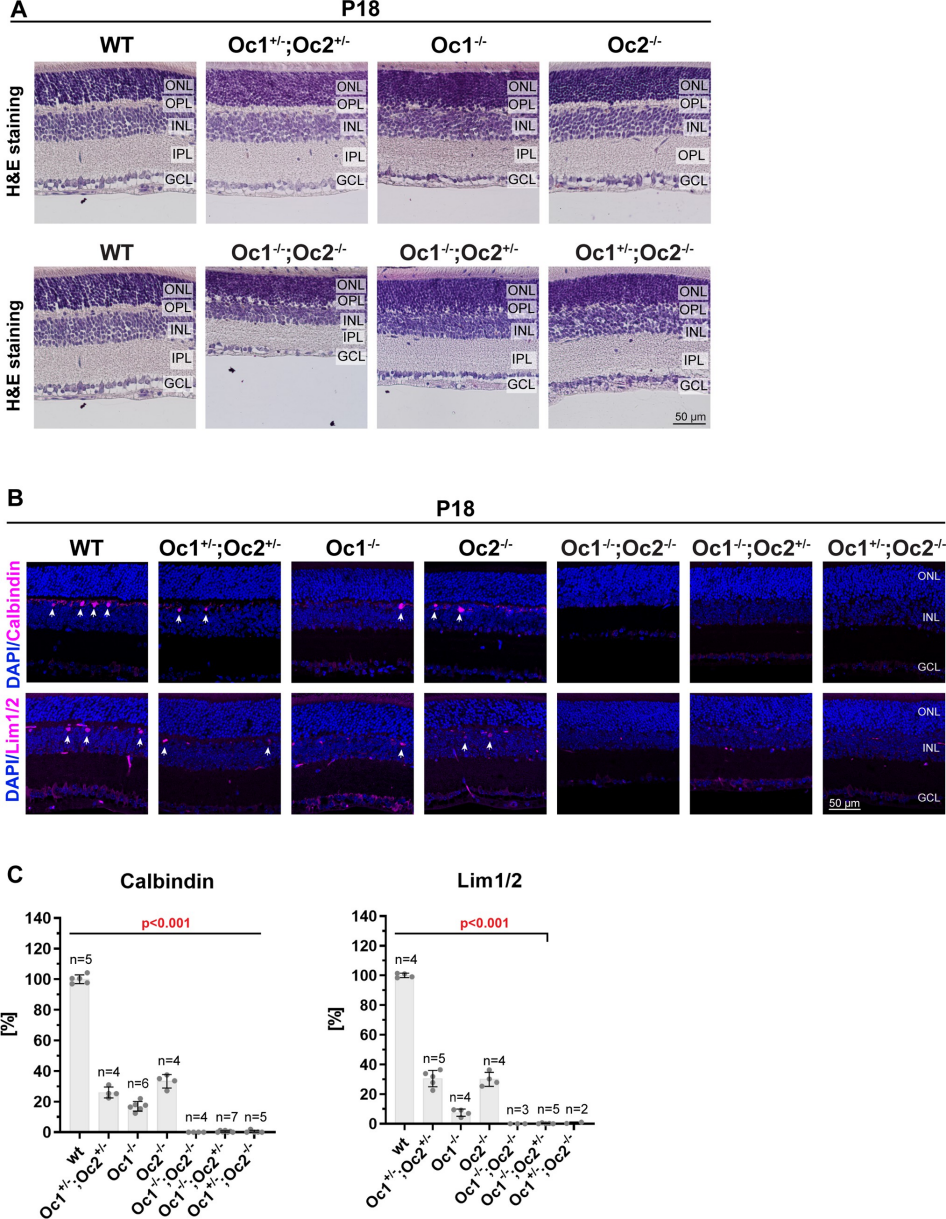
Reduction of OPL and decreased number of horizontal cells in P18 Oc-deficient mice. Eyes stained by H&E showed (A) reduced OPL in Oc-deficient mice retinas. Wt and Oc-deficient paraffin sections were stained (B) by horizontal cells marker calbindin and Lim1/2. Arrows indicate horizontal cells. Quantification (C) of number of horizontal cells stained by anti-calbindin and anti-Lim1/2 antibodies. Each point in graph represents value for one mouse. Cell counts were normalized to wt control. Mean frequency ± SD. Statistical significance was not determined for Oc1^+/-^; Oc2^-/-^ stained by Lim1/2 where only 2 mice were counted. GCL: ganglion cell layer; INL: inner nuclear layer; IPL: inner plexiform layer; ONL: outer nuclear layer; OPL: outer plexiform layer. Scale bar 50 μm.

To assess the phenotypic consequences of Oc1 and Oc2 deficiency, we investigated retinas at the histological level (Fig 1A). Retinal sections were acquired from mice at stage P18, when retinogenesis is complete. All retina-specific cell types are developed at this stage and mice have open eyes as eye opening occurs at P13-15 [40, 41]. Although all retinas displayed proper lamination, OPL localized between ONL and INL was reduced (Fig 1A). In Oc1^+/-^; Oc2^+/-^ and Oc2^-/-^ mice, OPL was slightly reduced and in Oc1^-/-^ mice was absent in most parts. In Oc1^-/-^; Oc2^-/-^, Oc1^-/-^; Oc2^+/-^ and Oc1^+/-^; Oc2^-/-^ animals, OPL was not even distinguishable, ONL fused with INL and formed one layer. Moreover, Oc1^-/-^; Oc2^-/-^ retinas had thinned INL, lower GCL density and slightly thinned ONL compared to the wild-type retina. IPL in Oc1^-/-^; Oc2^+/-^ was also partially reduced. Previous studies have shown that an Oc1^-/-^; Oc2^-/-^ retina has a decreased number of retinal cell types that are localized in the INL [11, 31] and thinned optic nerve compared to wild-type mouse [31].

These findings indicate that Oc proteins deficiency results in reduced OPL in the mouse retina. The level of reduction depends on the *Oc1/Oc2* genotype and thus on the level of the Oc proteins. The reduced OPL in Oc1^-/-^ and Oc2^-/-^ retinas and collapse of OPL in the Oc1^-/-^; Oc2^-/-^ retina are consistent with previous findings [7, 11, 31]. OPL is formed by HC and BC projections and photoreceptor terminals [7]. The reduction or even absence of this layer may indicate abolished HC and/or cone population, so we further examined these cell types by immunohistochemistry.

### HCs are sensitive to the dose of Oc proteins

To provide further insight into the phenotype of Oc-deficient retinas, we used immunohistochemistry to quantify the number of HCs that are localized between the ONL and INL. P18 retinal sections were stained with antibodies against calbindin and Lim1/2 (Fig 1B). The number of HCs was dramatically reduced in all Oc mutants (Fig 1C). HCs positive for calbindin staining decreased to 26% in double heterozygotes and were lost in mutants with one functional *Oc* allele, Oc1^-/-^; Oc2^+/-^ and Oc1^+/-^; Oc2^-/-^. The number of Lim1/2^+^ cells, which is another marker of HCs, decreased to 31% in double heterozygotes Oc1^+/-^; Oc2^+/-^ and Lim1/2^+^ cell were not detectable in mutants with only one functional *Oc1/2* allele. Dramatically reduced number of HCs in Oc1^-/-^ and Oc2^-/-^ mutants and loss of HCs in Oc1^-/-^; Oc2^-/-^ mouse retinas are consistent with current studies [7, 11, 31, 32].

Our data indicate that HCs are sensitive to the dose of Oc1 and Oc2 proteins. Half of the functional *Oc1/Oc2* alleles led to more than 50% decrease of HCs. The *Oc1/Oc2* double heterozygote was not able to compensate for the missing alleles and HCs were rapidly reduced according to the overall dose of Oc proteins. These findings implicate that a markedly reduced dose, or even absence, of Oc proteins leads to the loss of HCs in the mouse retina and that HCs are dependent on the dose of Oc proteins.

### Examination of other retinal cell types localized in the INL in Oc-deficient retinas

**Fig 2:**
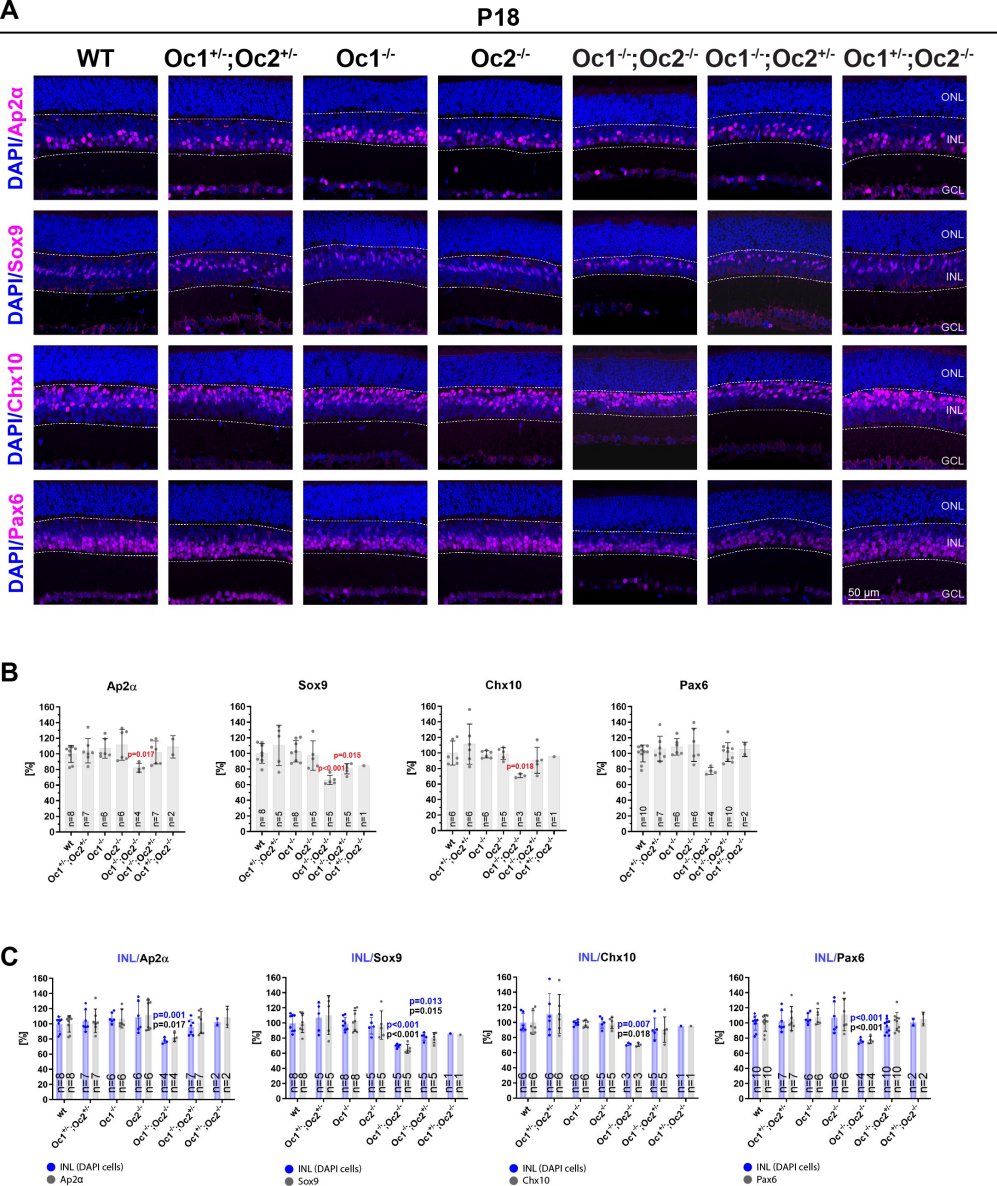
Changes in amacrine cells, Müller glial cells and bipolar cells in Oc mutants. Eye sections of P18 mice stained (A) by markers for amacrine cells (Ap2α), Müller glial cells (Sox9), bipolar cells (Chx10), and amacrine together with Müller glial cells (Pax6). Quantification (B) of Ap2α, Sox9, Chx10, and Pax6 positive cells. Each point in graph represents value for one mouse. Counts were normalized to wt control. Quantification (C) of DAPI-positive cells together with marker-positive cells in the INL. Each graph shows two columns for each genotype. Marker-positive cells are visualized as grey columns and DAPI-positive cells counted in the same eye sections as marker-positive cells are visualized as blue columns. Each point presented in graph represent value for one mouse. Eye sections were stained with the following markers: Ap2α, Sox9, Chx10 and Pax6. Counts of DAPI were normalized to DAPI-stained wt control. Cell counts of marker-positive cells were normalized to cell counts of marker-positive wt control. (B, C) Data information: mean frequency ± SD. Statistical significance was not determined in Oc1^+/-^; Oc2^-/-^ stained by anti-Ap2α, anti-Sox9, anti-Chx10 and anti-Pax6 antibodies, less than 3 mice were analyzed. GCL: ganglion cell layer; INL: inner nuclear layer; ONL: outer nuclear layer. Scale bar 50 μm.

Furthermore, we wanted to examine the consequences of Oc protein deficiency in other retinal cell types. We used immunohistochemistry to analyze individual retinal cell types localized in the INL (Fig 2A), in addition to HCs that were analyzed in Fig 1. The following markers were used: Ap2α for amacrine cells, Sox9 for Müller glia cells, Chx10 for bipolar cells and Pax6 for amacrine and Müller glial cells. Marker-positive cells were then quantified (Fig 2B). We did not notice statistically significant reduction of marker-positive cells in double heterozygote Oc1^+/-^; Oc2^+/-^. Mice with one functional *Oc2* allele (Oc1^-/-^; Oc2^+/-^) showed significantly reduced number of Sox9^+^ cells that decreased to 81%. Other marker-positive cells (Ap2α^+^, Chx10^+^, Pax6^+^) were not significantly decreased in Oc1^-/-^; Oc2^+/-^. Statistical significance was not assessed for mice with one functional *Oc1* allele (Oc1^+/-^; Oc2^-/-^) where less than three mice for each staining (Ap2α, Sox9, Chx10, Pax6) were analyzed. In agreement with previous studies [7, 31], significant reduction of marker-positive cells localized in INL, with exception of horizontal cells, was not observed in Oc1^-/-^ or Oc2^-/-^ mice. Statistically significant reduction of marker positive cells was observed in compound Oc1/2 double mutant mice (Oc1^-/-^; Oc2^-/-^). Ap2α^+^ cells decreased to 82%, Sox9^+^ cells to 66%, Chx10^+^ cells to 71%, and Pax6^+^ cells to 78%. H&E staining of compound Oc1/2 double mutant showed slightly thinner INL compared to the wt (Fig 1). Based on this observation, we also counted number of DAPI-positive cells localized in the INL. We counted DAPI-positive cells in wt and all generated *Oc*-deficient genotypes (Fig 2C). DAPI^+^ cells localized in the INL were counted and analyzed separately for each marker (Ap2α, Sox9, Chx10 and Pax6) and each genotype. DAPI^+^ cells were counted in the same eye sections as particular marker positive cells. DAPI-positive cells in Oc-deficient mice were normalized to DAPI-positive wt control and marker-positive cells were normalized to marker-positive wt control. We found that DAPI-positive cells were significantly reduced in Oc1^-/-^; Oc2^-/-^ mice and proportionally decreased to the similar level as marker-positive cells. DAPI-positive cells in the Oc1^-/-^; Oc2^-/-^ retina were decreased to 78% in Ap2α staining, to 70% in Sox9 staining, to 72% in Chx10 staining and to 77% in Pax6 staining. In Oc1^-/-^; Oc2^+/-^ mice, DAPI-positive cells were significantly reduced to 82% only in Sox9 staining. In this case, DAPI^+^ cells also decreased to similar level as marker positive cells. DAPI^+^ cell were not significantly decreased in double heterozygote Oc1^+/-^; Oc2^+/-^, in Oc1^-/-^ and Oc2^-/-^ mice. Oc1^+/-^; Oc2^-/-^ mice were not statistically evaluated as less than three mice per staining were analyzed. Based on these results, we further investigated whether proportions of Ap2α-, Sox9-, Chx10- and Pax6-positive cells in the INL changed in Oc-deficient mice (S1 Fig). Number of marker positive cells was normalized to number of DAPI-positive cells in the INL separately for each marker and genotype. Percentage of Ap2α^+^, Sox9^+^, Chx10^+^ and Pax6^+^ cells in the INL was not significantly affected in all studied mutant genotypes. Statistical analysis was not performed for Oc1^+/-^; Oc2^-/-^ mice.

Taken together, these results suggests that amacrine cells, Müller glial cells and bipolar cells were significantly decreased in the Oc1^-/-^; Oc2^-/-^ retina, but overall percentage representation of individual retinal cell types remained similar compared to wt mice.

### Cone photoreceptors and Tbr2^+^ retinal ganglion cells are influenced by deletion of Oc1 and Oc2

**Fig 3:**
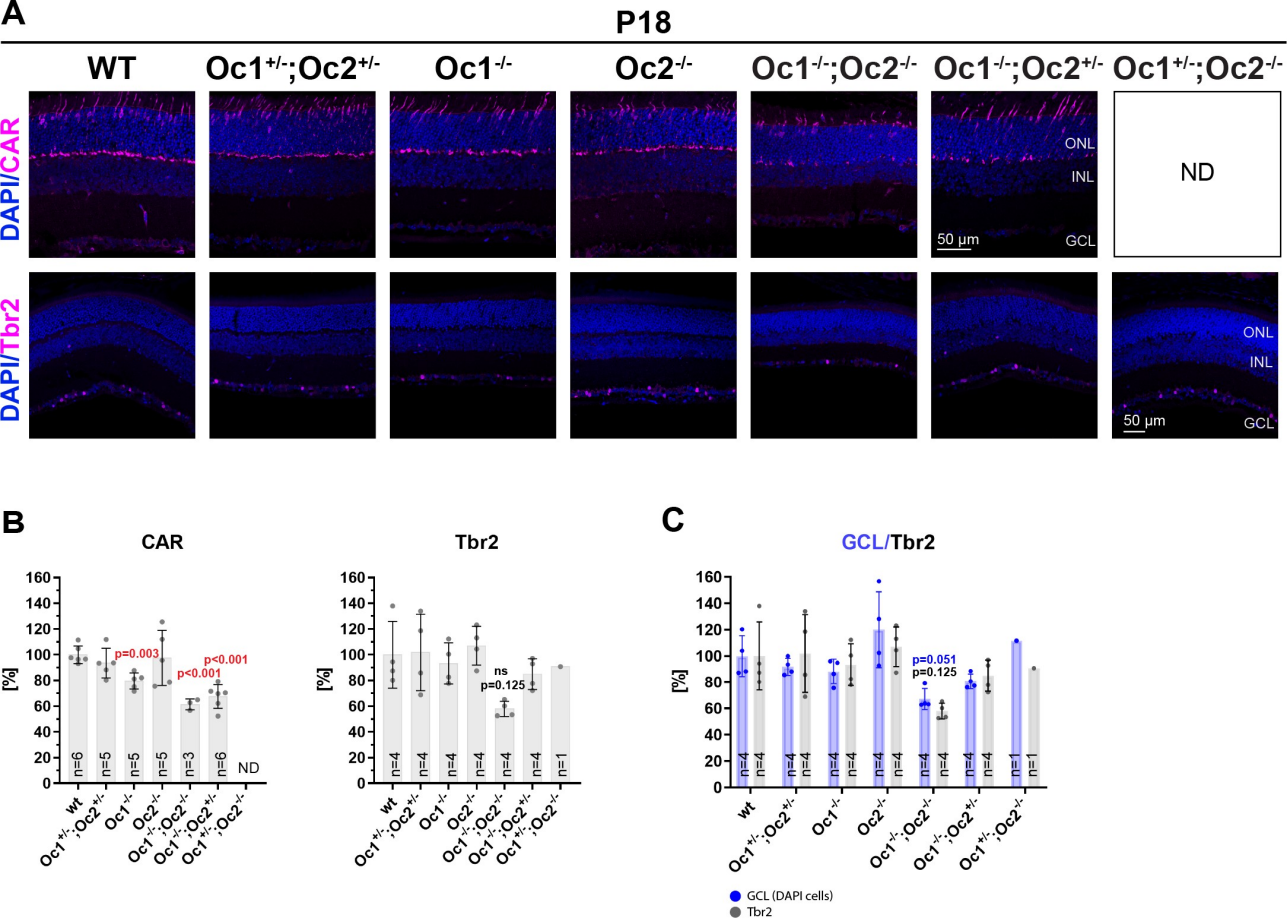
Decreased number of cones and retinal ganglion cells in Oc-deficient retina. P18 murine retinal sections stained by (A) cone arrestin (CAR), marker for cone photoreceptors and by Tbr2, marker for subset of RGCs (intrinsically photosensitive RGCs—ipRGCs). Quantification (B) of CAR and Tbr2-positive cells. Quantification (C) of DAPI-positive cells together with Tbr2-positive cells in the GCL. Tbr2- positive cells are presented as grey columns and DAPI-positive cells counted in the same retina sections as Tbr2 positive cells are visualized as blue columns. One point in the graph means value for one mouse. Cell counts were normalized to wt control. Counted DAPI-positive cells were normalized to DAPI-stained wt control. Tbr2-positive cell counts were normalized to Tbr2-stained positive wt control. Mean frequency ± SD. No significant difference between wt and Oc1^-/-^; Oc2^-/-^ is labeled ns. Statistical significance was not assessed for Oc1^+/-^; Oc2^-/-^ stained by Tbr2, only 2 mice were examined. ND: not determined, GCL: ganglion cell layer; INL: inner nuclear layer; ONL: outer nuclear layer. Scale bar 50 μm.

Oc1 and Oc2 are expressed during the retinal development in precursors of RGCs and cone photoreceptors [11, 29], and we therefore analyzed these cell types in P18 retinal sections by immunohistochemistry (Fig 3A). Cone photoreceptors staining using an antibody against cone arrestin and retinal ganglion cells were stained by anti-Tbr2 antibody. Tbr2 is expressed in a subset of RGCs and plays an important role in formation and maintenance of Opn4/melanopsin-expressing ipRGCs—intrinsically photosensitive retinal ganglion cells [42]. These cells constitute an important component of the non-image-forming visual system. The number of marker-positive cells was then quantified (Fig 3B). Cone photoreceptors detected by staining against cone arrestin were significantly reduced: in Oc1^-/-^ retina to 80%, in Oc1^-/-^; Oc2^-/-^ retina to 62% and in Oc1^-/-^; Oc2^+/-^ retina to 68%. Tbr2^+^ cells were markedly decreased only in case of Oc1^-/-^; Oc2^-/-^ to 58% (ns, p = 0,125). Quantification of DAPI-positive cells in the GCL (Fig 3C) revealed that DAPI-positive cells were reduced in Oc1^-/-^; Oc2^-/-^ to 67% (ns, p = 0,051). Tbr2^+^ cells normalized to DAPI cells in the GCL (S1 Fig) showed slight decrease without significance.

These data indicate, that cone photoreceptors were significantly reduced in Oc1^-/-^, Oc1^-/-^; Oc2^-/-^ and Oc1^-/-^; Oc2^+/-^ mice retinas. To investigate whether overall retinal function in Oc-deficient mice is compromised we performed ERG recording.

### Electroretinogram (ERG) recording

**Fig 4:**
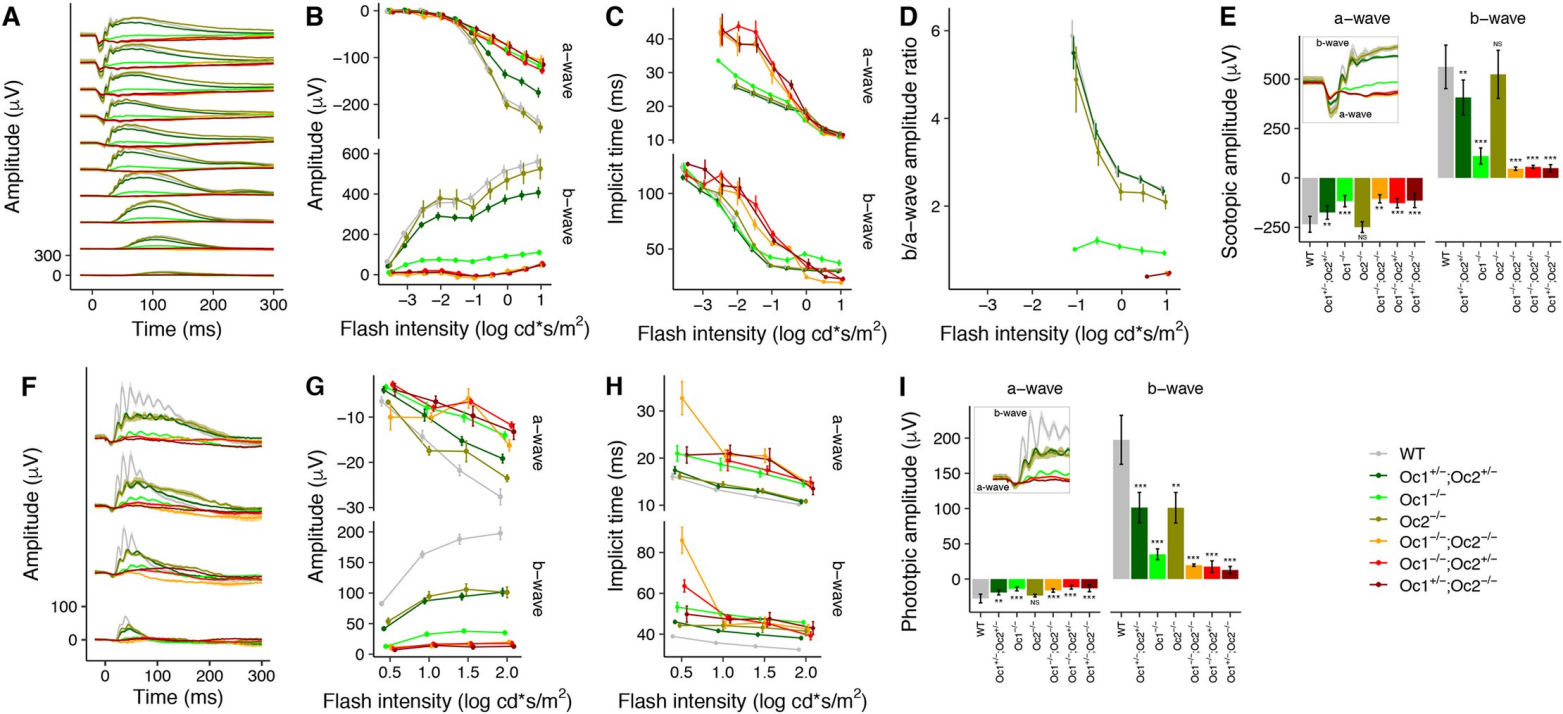
Cone and rod function deficits in Oc-deficient retina. In vivo recorded scotopic intensity-response functions (A) show a marked amplitude loss for all except Oc1+/-; Oc2+/- and Oc2-/- mice (B). Waveform implicit times are varying due to the loss of oscillations generated in the inner retina (C). This loss is best illustrated by the b-/a-wave amplitude ratio (D), where lower ratios correlate with the predominance of inner retinal function loss. Lowest ratios where found for Oc1^-/-^;Oc2^-/-^, Oc1^-/-^;Oc2^+/-^, Oc1^+/-^;Oc2^-/-^ mice. Detailed analysis for the highest flash intensity amplitudes (E) additionally shows a significant amplitude reduction for a- and b-wave amplitudes, indicating that photoreceptor and inner retinal functions are affected. Photopic single flash intensity-response functions (F) show consistent a-wave amplitude reductions except for Oc2-/- mice (G, I), for which a marked and significant b-wave amplitude reduction is found. The cone function is thus reduced in all Oc-deficient retinae.

For assessment of *in vivo* function, ERG-waveforms were recorded under scotopic (Fig 4A) and photopic (Fig 4D) conditions. Scotopic intensity-response functions show no effect in case of Oc2^-/-^ and a slight amplitude reduction of a- and b-waves in case of Oc1^+/-^; Oc2^+/-^ (Fig 4F), but a similarly distinct loss of b-wave amplitudes under photopic conditions (Fig 4E). However, a-wave amplitudes, corresponding to the cone photoreceptor function, show only a mild amplitude reduction. For both, rod and cone function, a marked decrease in the ERG signal was observed in Oc1^-/-^, Oc1^-/-^; Oc2^-/-^, Oc1^-/-^; Oc2^+/-^ and Oc1^+/-^; Oc2^-/-^ mice. Additionally, in all Oc-deficient mice the a-wave was better preserved than the b-wave, consistent with a predominant dysfunction of the inner retina (primarily BCs). Interestingly, although the amplitudes were markedly reduced, there was little variation in the waveform component implicit times under scotopic as well as photopic conditions. This finding may be explained by a reduced cell number but almost normal cell function. Consistent results were found for photopic flicker responses (S2 Fig).

## Discussion

HCs represent a small part of the retinal cell population, only about 0.2% [43], but they have a very important function. Horizontal interneurons provide a negative feedback to photoreceptors and connection to bipolar cells [44, 45] and are necessary to maintain the working range at different illumination levels. We investigated Oc-deficient mice in which a decreased number of HCs corresponded to the *Oc1/Oc2* genotype and thus to the dose of Oc1/Oc2 proteins. The level of reduction of HCs corresponds to the extent of thickness reduction of the INL containing the cell bodies and OPL where the synapses of HCs are located. Mice with a markedly reduced number, or even absence of HCs had markedly reduced thickness of, or even absent OPL. HCs synapsing with photoreceptors in OPL are required for their survival. It was shown that the loss of the majority of HCs leads to an age-dependent degeneration of photoreceptors in *Oc1*^*-/-*^ mice [7]. OPL disappeared at the age of 5 months and ONL was markedly thinned, being only one-third thickness of the wild-type at 8 months, indicating disrupted survival of photoreceptors. Scotopic ERG recording of 5-month-old mice confirmed the reduced photoreceptor function in *Oc1*^*-/-*^ mice [7]. Ablation of HCs using the diphtheria toxin receptor system in adult mouse retinas leads to a significantly decreased number of photoreceptor nuclei, disappearing OPL, thinning of the retina and a marked reduction of the scotopic ERG bipolar cell component and an almost absent photopic ERG [46]. These findings point out the important role of HCs in photoreceptor survival. The number of horizontal interneurons is dependent on the dose of Oc proteins, which are also expressed in the cone precursors during development [11, 29]. A previous study discovered that Oc1^-/-^; Oc2^-/-^ mice failed in cone genesis at E14.5; nevertheless, cones were developed at E17.5, but their total number was decreased by 30%. This indicated that cones are developed in two ways, one that is dependent on Oc transcription factors and one that is either compensatory or completely independent of them [31]. In accordance with the decreased number of cone photoreceptors at E17.5 in double mutant mice, we observed a significant reduction of cone numbers, by 39%, in Oc1^-/-^; Oc2^-/-^ mice at P18. In Oc1^-/-^; Oc2^+/-^ mice at P18, the cone numbers were significantly reduced, by 32%. Other studies showed reduction of cone photoreceptors in double mutants by 23% in P18 mice [11], by 34% in P5 mice, with a reduction of 30% persisting in adulthood [31]. Cones in other Oc-deficient genotypes were not significantly influenced. This could be explained by the fact that there exists a threshold dose of Oc proteins required for the proper cone genesis during development.

Gene duplication represents a major mechanism through which new genetic material is generated during molecular evolution. Ancestral vertebrate genome has undergone two rounds of whole genome duplication [47] leading to the emergence of paralogs that were essentially free to undergo the process of neofunctionalization, subfunctionalization, or degeneration (gene loss) [48]. Genetic redundancy created by duplication enables neofunctionalization of one of the gene duplicates that can undergo a series of coding or regulatory mutations without harming the vitality of the organism since the other copy preserves the original function. In contrast, during the subfunctionalization process both gene copies can accumulate degenerative mutations provided that any defects in one gene copy are complemented by the other copy. Duplication-degeneration-complementation model proposes that the functionality of the original gene is distributed among the two copies, neither of which can be lost, as they perform non-redundant functions [49]. Last but not least, some duplications may be lost as they are either detrimental or not beneficial to the host organism. Our study of the functional role of two paralogous genes, Oc1 and Oc2, for retina anatomy, cell composition and function revealed an unexpected gene interaction. We found that in the context of HCs development, the four Oc1/Oc2 alleles appear equivalent and redundant, as similar defects are obtained in *Oc1*^*-/-*^, *Oc2*^*-/-*^ and *Oc1*^*+/-*^*; Oc2*^*+/-*^ double heterozygote mice. This is in contrast to Oc1/Oc2 alleles non-redundant function in other tissues like pancreas and endocrine system [50]. In addition, a single functional allele of either Oc1 or Oc2 (in the presence of a complete knockout of the other paralogue) is not able to support HCs development indicating strong dosage requirement. Although we cannot exclude that Oc1/Oc2 paralogs could have other, more or less divergent functions not adressed in our study, we conclude that Oc1/Oc2 paralogous genes function additively to support HCs fate and so their molecular evolution does not follow one of the typical routes after gene duplication.

## Supporting information

S1 Fig Presence of individual retinal cell types in the INL and GCL in Oc-deficient mice. Counts of Ap2α, Sox9, Chx10, and Pax6 positive cells were normalized to counts of DAPI-positive cells in the INL and counts of Tbr2 positive cells were normalized to counts of DAPI-positive cells in the GCL. Marker positive and DAPI^+^ cells were counted in the same sections, separately for each genotype. One point in graph means value for one mouse. Mean frequency ± SD. Statistical significance was not determined for Oc1^+/-^; Oc2^-/-^ where less than 3 mice were analyzed for each staining.
(TIF)

S2 Fig Cone function in Oc-deficient retina. A magnitude loss is found for the responses to flickering lights (A, B). Interestingly, the bandpass characteristics of the flicker frequency-response function (B) is almost unchanged. Additionally, we found phase (C) changes that may be attributed to the loss of oscillations generated in the inner retina.
(JPG)

## References

[1] Cepko CL, Austin CP, Yang X, Alexiades M, Ezzeddine D. Cell fate determination in the vertebrate retina. Proc Natl Acad Sci U S A. 1996;93(2):589–95. Epub 1996/01/23. 10.1073/pnas.93.2.589 8570600 PMC40096

[2] Marquardt T, Ashery-Padan R, Andrejewski N, Scardigli R, Guillemot F, Gruss P. Pax6 is required for the multipotent state of retinal progenitor cells. Cell. 2001;105(1):43–55. Epub 2001/04/13. 10.1016/s0092-8674(01)00295-1 . 11301001

[3] Ohsawa R, Kageyama R. Regulation of retinal cell fate specification by multiple transcription factors. Brain Res. 2008;1192:90–8. Epub 2007/05/10. 10.1016/j.brainres.2007.04.014 . 17488643

[4] Cepko C. Intrinsically different retinal progenitor cells produce specific types of progeny. Nature reviews Neuroscience. 2014;15(9):615–27. Epub 2014/08/07. 10.1038/nrn3767 . 25096185

[5] Heavner W, Pevny L. Eye development and retinogenesis. Cold Spring Harb Perspect Biol. 2012;4(12). Epub 2012/10/17. 10.1101/cshperspect.a008391 23071378 PMC3504437

[6] Wassle H, Boycott BB. Functional architecture of the mammalian retina. Physiol Rev. 1991;71(2):447–80. Epub 1991/04/01. 10.1152/physrev.1991.71.2.447 . 2006220

[7] Wu F, Li R, Umino Y, Kaczynski TJ, Sapkota D, Li S, et al Onecut1 is essential for horizontal cell genesis and retinal integrity. J Neurosci. 2013;33(32):13053–65, 65a. Epub 2013/08/09. 10.1523/JNEUROSCI.0116-13.2013 23926259 PMC3735885

[8] Dedek K, Breuninger T, de Sevilla Muller LP, Maxeiner S, Schultz K, Janssen-Bienhold U, et al A novel type of interplexiform amacrine cell in the mouse retina. Eur J Neurosci. 2009;30(2):217–28. Epub 2009/07/21. 10.1111/j.1460-9568.2009.06808.x . 19614986

[9] Cepko CL. The roles of intrinsic and extrinsic cues and bHLH genes in the determination of retinal cell fates. Curr Opin Neurobiol. 1999;9(1):37–46. Epub 1999/03/11. 10.1016/s0959-4388(99)80005-1 . 10072376

[10] Xiang M. Intrinsic control of mammalian retinogenesis. Cell Mol Life Sci. 2013;70(14):2519–32. Epub 2012/10/16. 10.1007/s00018-012-1183-2 23064704 PMC3566347

[11] Klimova L, Antosova B, Kuzelova A, Strnad H, Kozmik Z. Onecut1 and Onecut2 transcription factors operate downstream of Pax6 to regulate horizontal cell development. Dev Biol. 2015;402(1):48–60. Epub 2015/03/22. 10.1016/j.ydbio.2015.02.023 . 25794677

[12] Livesey FJ, Cepko CL. Vertebrate neural cell-fate determination: lessons from the retina. Nature reviews Neuroscience. 2001;2(2):109–18. Epub 2001/03/17. 10.1038/35053522 . 11252990

[13] Young RW. Cell differentiation in the retina of the mouse. The Anatomical record. 1985;212(2):199–205. Epub 1985/06/01. 10.1002/ar.1092120215 . 3842042

[14] Klimova L, Kozmik Z. Stage-dependent requirement of neuroretinal Pax6 for lens and retina development. Development (Cambridge, England). 2014;141(6):1292–302. Epub 2014/02/14. 10.1242/dev.098822 . 24523460

[15] Farhy C, Elgart M, Shapira Z, Oron-Karni V, Yaron O, Menuchin Y, et al Pax6 is required for normal cell-cycle exit and the differentiation kinetics of retinal progenitor cells. PLoS One. 2013;8(9):e76489 Epub 2013/09/28. 10.1371/journal.pone.0076489 24073291 PMC3779171

[16] Jacquemin P, Lannoy VJ, Rousseau GG, Lemaigre FP. OC-2, a novel mammalian member of the ONECUT class of homeodomain transcription factors whose function in liver partially overlaps with that of hepatocyte nuclear factor-6. The Journal of biological chemistry. 1999;274(5):2665–71. Epub 1999/01/23. 10.1074/jbc.274.5.2665 . 9915796

[17] Lannoy VJ, Bürglin TR, Rousseau GG, Lemaigre FP. Isoforms of hepatocyte nuclear factor-6 differ in DNA-binding properties, contain a bifunctional homeodomain, and define the new ONECUT class of homeodomain proteins. The Journal of biological chemistry. 1998;273(22):13552–62. Epub 1998/06/05. 10.1074/jbc.273.22.13552 . 9593691

[18] Vanhorenbeeck V, Jacquemin P, Lemaigre FP, Rousseau GG. OC-3, a novel mammalian member of the ONECUT class of transcription factors. Biochem Biophys Res Commun. 2002;292(4):848–54. Epub 2002/04/12. 10.1006/bbrc.2002.6760 . 11944891

[19] Landry C, Clotman F, Hioki T, Oda H, Picard JJ, Lemaigre FP, et al HNF-6 is expressed in endoderm derivatives and nervous system of the mouse embryo and participates to the cross-regulatory network of liver-enriched transcription factors. Dev Biol. 1997;192(2):247–57. Epub 1998/01/27. 10.1006/dbio.1997.8757 . 9441665

[20] Jacquemin P, Durviaux SM, Jensen J, Godfraind C, Gradwohl G, Guillemot F, et al Transcription factor hepatocyte nuclear factor 6 regulates pancreatic endocrine cell differentiation and controls expression of the proendocrine gene ngn3. Mol Cell Biol. 2000;20(12):4445–54. Epub 2000/05/29. 10.1128/mcb.20.12.4445-4454.2000 10825208 PMC85812

[21] Clotman F, Lannoy VJ, Reber M, Cereghini S, Cassiman D, Jacquemin P, et al The onecut transcription factor HNF6 is required for normal development of the biliary tract. Development (Cambridge, England). 2002;129(8):1819–28. Epub 2002/04/06. . 11934848 10.1242/dev.129.8.1819

[22] Bouzin C, Clotman F, Renauld JC, Lemaigre FP, Rousseau GG. The onecut transcription factor hepatocyte nuclear factor-6 controls B lymphopoiesis in fetal liver. J Immunol. 2003;171(3):1297–303. Epub 2003/07/23. 10.4049/jimmunol.171.3.1297 . 12874218

[23] Clotman F, Jacquemin P, Plumb-Rudewiez N, Pierreux CE, Van der Smissen P, Dietz HC, et al Control of liver cell fate decision by a gradient of TGF beta signaling modulated by Onecut transcription factors. Genes Dev. 2005;19(16):1849–54. Epub 2005/08/17. 10.1101/gad.340305 16103213 PMC1186184

[24] Francius C, Clotman F. Dynamic expression of the Onecut transcription factors HNF-6, OC-2 and OC-3 during spinal motor neuron development. Neuroscience. 2010;165(1):116–29. Epub 2009/10/06. 10.1016/j.neuroscience.2009.09.076 . 19800948

[25] Audouard E, Schakman O, Ginion A, Bertrand L, Gailly P, Clotman F. The Onecut transcription factor HNF-6 contributes to proper reorganization of Purkinje cells during postnatal cerebellum development. Mol Cell Neurosci. 2013;56:159–68. Epub 2013/05/15. 10.1016/j.mcn.2013.05.001 . 23669529

[26] Lemaigre FP, Durviaux SM, Truong O, Lannoy VJ, Hsuan JJ, Rousseau GG. Hepatocyte nuclear factor 6, a transcription factor that contains a novel type of homeodomain and a single cut domain. Proc Natl Acad Sci U S A. 1996;93(18):9460–4. 10.1073/pnas.93.18.9460 . 8790352 PMC38450

[27] Tan Y, Yoshida Y, Hughes DE, Costa RH. Increased expression of hepatocyte nuclear factor 6 stimulates hepatocyte proliferation during mouse liver regeneration. Gastroenterology. 2006;130(4):1283–300. Epub 2006/04/19. 10.1053/j.gastro.2006.01.010 16618419 PMC1440887

[28] Jacquemin P, Durviaux SM, Jensen J, Godfraind C, Gradwohl G, Guillemot F, et al Transcription factor hepatocyte nuclear factor 6 regulates pancreatic endocrine cell differentiation and controls expression of the proendocrine gene ngn3. Mol Cell Biol. 2000;20(12):4445–54. 10.1128/mcb.20.12.4445-4454.2000 . 10825208 PMC85812

[29] Wu F, Sapkota D, Li R, Mu X. Onecut 1 and Onecut 2 are potential regulators of mouse retinal development. J Comp Neurol. 2012;520(5):952–69. Epub 2011/08/11. 10.1002/cne.22741 21830221 PMC3898336

[30] Sapkota D, Mu X. Onecut transcription factors in retinal development and maintenance. Neural Regen Res. 2015;10(6):899–900. Epub 2015/07/23. 10.4103/1673-5374.158350 26199604 PMC4498349

[31] Sapkota D, Chintala H, Wu F, Fliesler SJ, Hu Z, Mu X. Onecut1 and Onecut2 redundantly regulate early retinal cell fates during development. Proc Natl Acad Sci U S A. 2014;111(39):E4086–95. Epub 2014/09/18. 10.1073/pnas.1405354111 25228773 PMC4191802

[32] Goetz JJ, Martin GM, Chowdhury R, Trimarchi JM. Onecut1 and Onecut2 play critical roles in the development of the mouse retina. PLoS One. 2014;9(10):e110194 Epub 2014/10/15. 10.1371/journal.pone.0110194 25313862 PMC4196951

[33] Emerson MM, Surzenko N, Goetz JJ, Trimarchi J, Cepko CL. Otx2 and Onecut1 promote the fates of cone photoreceptors and horizontal cells and repress rod photoreceptors. Dev Cell. 2013;26(1):59–72. Epub 2013/07/23. 10.1016/j.devcel.2013.06.005 23867227 PMC3819454

[34] Li S, Mo Z, Yang X, Price SM, Shen MM, Xiang M. Foxn4 controls the genesis of amacrine and horizontal cells by retinal progenitors. Neuron. 2004;43(6):795–807. Epub 2004/09/15. 10.1016/j.neuron.2004.08.041 . 15363391

[35] Fujitani Y, Fujitani S, Luo H, Qiu F, Burlison J, Long Q, et al Ptf1a determines horizontal and amacrine cell fates during mouse retinal development. Development (Cambridge, England). 2006;133(22):4439–50. Epub 2006/11/01. 10.1242/dev.02598 . 17075007

[36] Klimova L, Lachova J, Machon O, Sedlacek R, Kozmik Z. Generation of mRx-Cre transgenic mouse line for efficient conditional gene deletion in early retinal progenitors. PLoS One. 2013;8(5):e63029 Epub 2013/05/15. 10.1371/journal.pone.0063029 23667567 PMC3646923

[37] Zhang H, Ables ET, Pope CF, Washington MK, Hipkens S, Means AL, et al Multiple, temporal-specific roles for HNF6 in pancreatic endocrine and ductal differentiation. Mech Dev. 2009;126(11–12):958–73. Epub 2009/09/22. 10.1016/j.mod.2009.09.006 19766716 PMC2783291

[38] Leopold SA, Zeilbeck LF, Weber G, Seitz R, Bosl MR, Jagle H, et al Norrin protects optic nerve axons from degeneration in a mouse model of glaucoma. Scientific reports. 2017;7(1):14274 Epub 2017/10/29. 10.1038/s41598-017-14423-8 29079753 PMC5660254

[39] Mages K, Grassmann F, Jagle H, Rupprecht R, Weber BHF, Hauck SM, et al The agonistic TSPO ligand XBD173 attenuates the glial response thereby protecting inner retinal neurons in a murine model of retinal ischemia. Journal of neuroinflammation. 2019;16(1):43 Epub 2019/02/20. 10.1186/s12974-019-1424-5 30777091 PMC6378755

[40] Ko H, Cossell L, Baragli C, Antolik J, Clopath C, Hofer SB, et al The emergence of functional microcircuits in visual cortex. Nature. 2013;496(7443):96–100. Epub 2013/04/05. 10.1038/nature12015 23552948 PMC4843961

[41] Yates D. Visual processing: eye-opening reorganization. Nature reviews Neuroscience. 2013;14(5):309 Epub 2013/04/20. 10.1038/nrn3501 . 23598725

[42] Mao C-A, Li H, Zhang Z, Kiyama T, Panda S, Hattar S, et al T-box transcription regulator Tbr2 is essential for the formation and maintenance of Opn4/melanopsin-expressing intrinsically photosensitive retinal ganglion cells. J Neurosci. 2014;34(39):13083–95. 10.1523/JNEUROSCI.1027-14.2014 . 25253855 PMC4172803

[43] Ajioka I, Martins RA, Bayazitov IT, Donovan S, Johnson DA, Frase S, et al Differentiated horizontal interneurons clonally expand to form metastatic retinoblastoma in mice. Cell. 2007;131(2):378–90. Epub 2007/10/25. 10.1016/j.cell.2007.09.036 17956737 PMC2203617

[44] Masland RH. The neuronal organization of the retina. Neuron. 2012;76(2):266–80. 10.1016/j.neuron.2012.10.002 23083731 PMC3714606

[45] Poche RA, Reese BE. Retinal horizontal cells: challenging paradigms of neural development and cancer biology. Development (Cambridge, England). 2009;136(13):2141–51. Epub 2009/06/09. 10.1242/dev.033175 19502480 PMC2729336

[46] Sonntag S, Dedek K, Dorgau B, Schultz K, Schmidt KF, Cimiotti K, et al Ablation of retinal horizontal cells from adult mice leads to rod degeneration and remodeling in the outer retina. J Neurosci. 2012;32(31):10713–24. Epub 2012/08/03. 10.1523/JNEUROSCI.0442-12.2012 . 22855819 PMC6621400

[47] Dehal P, Boore JL. Two rounds of whole genome duplication in the ancestral vertebrate. PLoS Biol. 2005;3(10):e314 10.1371/journal.pbio.0030314 16128622 PMC1197285

[48] Taylor JS, Raes J. Duplication and divergence: the evolution of new genes and old ideas. Annu Rev Genet. 2004;38:615–43. 10.1146/annurev.genet.38.072902.092831 . 15568988

[49] Force A, Lynch M, Pickett FB, Amores A, Yan YL, Postlethwait J. Preservation of duplicate genes by complementary, degenerative mutations. Genetics. 1999;151(4):1531–45. 10101175 10.1093/genetics/151.4.1531 PMC1460548

[50] Vanhorenbeeck V, Jenny M, Cornut J-F, Gradwohl G, Lemaigre FP, Rousseau GG, et al Role of the Onecut transcription factors in pancreas morphogenesis and in pancreatic and enteric endocrine differentiation. Developmental Biology. 2007;305(2):685–94. 10.1016/j.ydbio.2007.02.027 17400205

