# Supplementary figures and images for: Dose-dependent regulation of horizontal cell fate by Onecut family of transcription factors
Source: PLoS One. 2020 Aug 13;15(8):e0237403. doi: 10.1371/journal.pone.0237403 (PMC7425962; doi:10.1371/journal.pone.0237403)

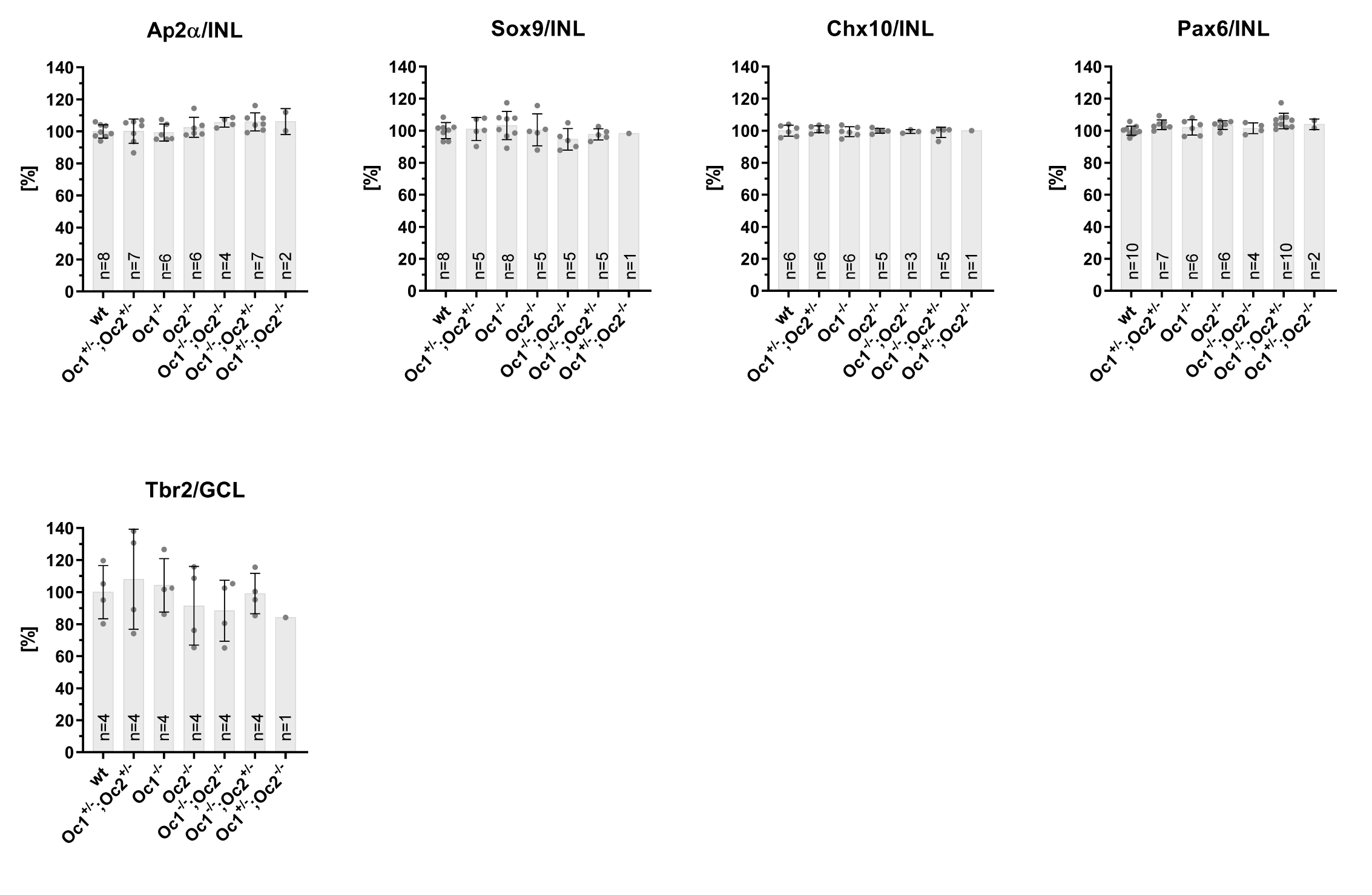

Supplement: S1 Fig — Counts of Ap2α, Sox9, Chx10, and Pax6 positive cells were normalized to counts of DAPI-positive cells in the INL and counts of Tbr2 positive cells were normalized to counts of DAPI-positive cells in the GCL. Marker positive and DAPI+ cells were counted in the same sections, separately for each genotype. One point in graph means value for one mouse. Mean frequency ± SD. Statistical significance was not determined for Oc1+/-; Oc2-/- where less than 3 mice were analyzed for each staining. (TIF) [file pone.0237403.s001.tif]

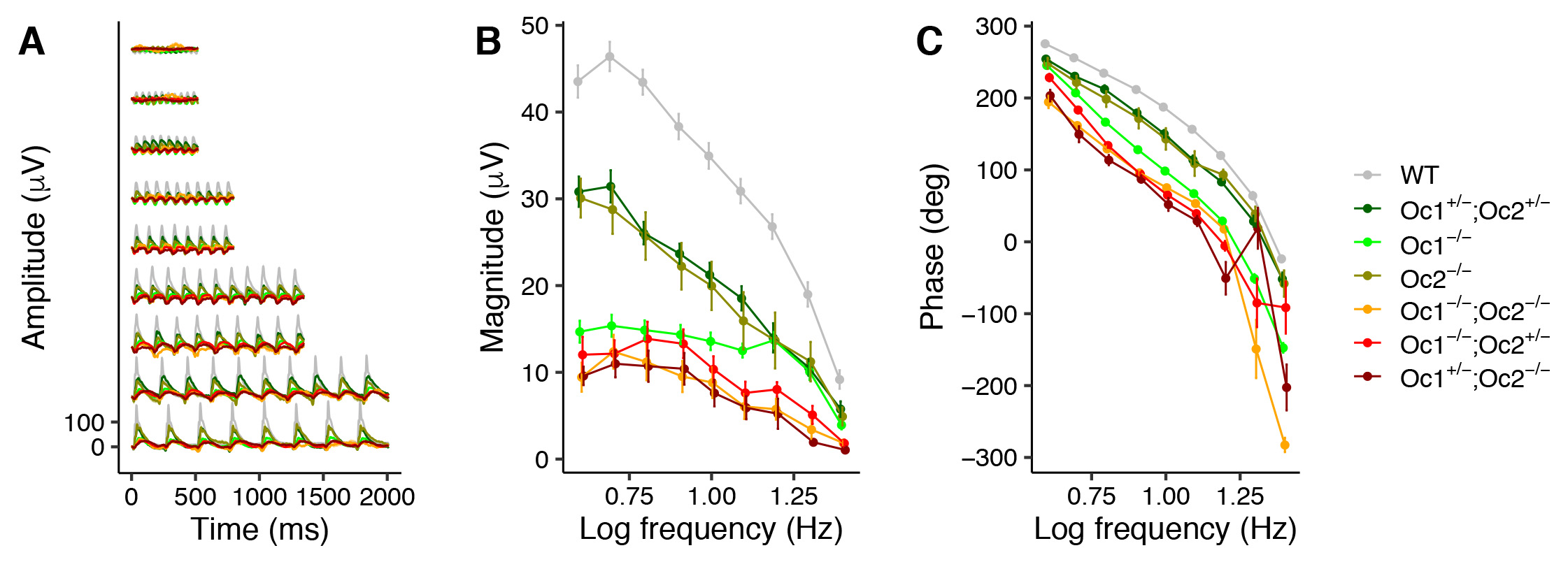

Supplement: S2 Fig — A magnitude loss is found for the responses to flickering lights (A, B). Interestingly, the bandpass characteristics of the flicker frequency-response function (B) is almost unchanged. Additionally, we found phase (C) changes that may be attributed to the loss of oscillations generated in the inner retina. (JPG) [file pone.0237403.s002.jpg]
